# Enhancing the value of accelerometer-assessed physical activity: meaningful visual comparisons of data-driven translational accelerometer metrics

**DOI:** 10.1186/s40798-019-0225-9

**Published:** 2019-12-05

**Authors:** Alex V. Rowlands, Nathan P. Dawkins, Ben Maylor, Charlotte L. Edwardson, Stuart J. Fairclough, Melanie J. Davies, Deirdre M. Harrington, Kamlesh Khunti, Tom Yates

**Affiliations:** 1 Diabetes Research Centre, Leicester General Hospital, University of Leicester, Leicester, LE5 4PW UK; 2 NIHR Leicester Biomedical Research Centre, Leicester, UK; 3 Alliance for Research in Exercise, Nutrition and Activity (ARENA), Sansom Institute for Health Research, Division of Health Sciences, University of South Australia, Adelaide, Australia; 4 Movement Behaviours, Health, and Wellbeing Research Group, Department of Sport and Physical Activity, Edge Hill University, Ormskirk, UK; 5 NIHR Collaboration for Leadership in Applied Health Research and Care East Midlands, Leicester General Hospital, Leicester, UK

## Abstract

The lack of consensus on meaningful and interpretable physical activity outcomes from accelerometer data hampers comparison across studies. Cut-point analyses are simple to apply and easy to interpret but can lead to results that are not comparable. We propose that the optimal accelerometer metrics for data analysis are not the same as the optimal metrics for translation. Ideally, analytical metrics are precise continuous variables that cover the intensity spectrum, while translational metrics facilitate meaningful, public-health messages and can be described in terms of activities (e.g. brisk walking) or intensity (e.g. moderate-to-vigorous physical activity). Two analytical metrics that capture the volume and intensity of the 24-h activity profile are average acceleration (volume) and intensity gradient (intensity distribution). These allow investigation of independent, additive and interactive associations of volume and intensity of activity with health; however, they are not immediately interpretable. The MX metrics, the acceleration above which the most active X minutes are accumulated, are translational metrics that can be interpreted in terms of indicative activities. Using a range of MX metrics illustrates the intensity gradient and average acceleration (i.e. 24-h activity profile). The M120, M60, M30, M15 and M5 illustrate the most active accumulated minutes of the day, the M^1^/_3DAY_ the most active accumulated 8 h of the day. We demonstrate how radar plots of MX metrics can be used to interpret and translate results from between- and within-group comparisons, provide information on meeting guidelines, assess individual activity profiles relative to percentiles and compare activity profiles between domains and/or time periods.

## Key Points

- For accelerometer-assessed physical activity, the optimal analytical metrics are precise, cover the whole spectrum of physical activity intensities and reflect directly measured acceleration. The optimal translational metrics are associated with greater prediction error as they need to be expressed in terms of typical activities (e.g. brisk walking, running).
- The average acceleration and intensity gradient are continuous data-driven analytical metrics that facilitate investigation of independent, additive and interactive effects of volume and intensity of physical activity on health.
- Radar plots of the MX translational metrics provide a visual translation of these analyses, in relation to typical activities (e.g. walking, running), facilitating the development of public-health friendly recommendations.

## Main text

### *The problem*

Large-scale studies of accelerometer-measured physical activity have become commonplace. However, the lack of consensus on meaningful and interpretable activity outcomes hampers comparison or data harmonization across studies, limiting the potential value of these data. When accelerometers started being used in physical activity research, in order to give biological meaning to the data, cut-points were developed to calibrate accelerometer output relative to energy expenditure [1]. Application of cut-points allowed estimates of time spent at a given activity intensity, e.g. time spent in moderate-to-vigorous physical activity (MVPA). Cut-point approaches are commonly used as they are simple to apply, lend themselves to interpretation relative to public health guidelines and have facilitated substantial progress in the evaluation of physical activity in relation to health (e.g. [2, 3]). However, application of cut-points collapses data into categories for analysis, rendering it impossible to compare datasets deploying different cut-points. This is problematic as multiple cut-points are available and in regular use [4, 5]. Recently Migueles et al. [5] demonstrated that the prevalence of meeting guidelines varied from 8 to 96% of their sample of children depending on which cut-point they applied. They concluded that it was ‘not possible (and probably will never be) to know the prevalence of meeting the physical activity guidelines based on accelerometer data’ ([5], p., 1). One approach to address this has been the development of conversion equations to ‘standardise’ estimates of MVPA to a common cut-point estimate [4]. A further problem is that, by definition, collapsing data into categories by applying cut-points prior to analysis leads to results that ignore substantial proportions of the data.

### *Separating the analysis and translation of accelerometer data*

We propose that the accelerometer metrics used for data analysis do not have to be the same as the metrics used for translation or interpretation. Indeed, the requirements for optimal analytical metrics (e.g. to assess associations with health or effectiveness of interventions) and for optimal translational metrics (e.g. to translate results into activity recommendations) can be contradictory. For example, analytical accelerometer metrics ideally cover the whole spectrum of physical activity intensities and reflect directly measured acceleration; the further the accelerometer metric moves from the original dimension measured, i.e. acceleration, the greater the scope for error in the estimation of the physical activity dimension [6]. In contrast, to be meaningful, physical activity recommendations need to be couched in terms of discrete recognisable activities or intensities, e.g. walking, running and/or moderate-to-vigorous physical activity (MVPA), that typically only account for relatively small proportions of the day. As these are not the directly measured variables, and there is variability in accelerometer outcomes for any given activity type or intensity, prediction of these activities or intensities increases error [6]. Arguably, this is more acceptable for translation where examples of activity types are given than for analysis where greater precision of measurement is required. Precision is necessary to increase the specificity of PA exposures for specific health outcomes, understanding of dose-response relationships, sensitivity to the effects of interventions, and to monitor temporal trends in physical activity [7].

We propose that using different metrics for analysis and for translation of accelerometer data could help facilitate comparison between datasets using the same accelerometer wear-site while maintaining meaningful translation of accelerometer data.

#### Analytical metrics

There is increasing recognition of the importance of capturing the intensity distribution of physical activity rather than focusing on MVPA alone (e.g. [8]). Specifically, it is important to capture both volume and the intensity distribution as, depending on the health outcome/marker, the volume of activity may be more important than the intensity (e.g. [9–11]), intensity may be key (e.g. [12, 13]) or intensity and volume may have a cumulative effect (e.g. [14]). Two data-driven metrics that, together, capture the volume and intensity of physical activity of the 24-h activity profile are the average acceleration (indicative of volume) and the intensity gradient (intensity distribution) [15]. In brief, the intensity gradient describes the negative curvilinear relationship between activity intensity and the time accumulated at that intensity. The intensity gradient is always negative, reflecting the drop in time accumulated as intensity increases; a more negative (lower) gradient reflects a steeper drop with little time accumulated at mid-range and higher intensities, while a less negative (higher) gradient reflects a shallower drop with more time spread across the intensity range. Together, the average acceleration and the intensity gradient enable investigation into whether the volume and intensity of physical activity have independent, additive or interactive effects on health. Using these metrics, we have recently shown that the intensity gradient was independently associated with body fatness, metabolic risk and cardiorespiratory fitness in children [14, 16]; intensity and volume were additively associated with body fat in adults; and adult bone health was highest if activity intensity was high, somewhat irrespective of volume [14].

While appropriate for data analyses due to coverage of the entire 24-h period and not imposing arbitrary cut-points on data, these metrics are not immediately interpretable or public-health message friendly. The purpose of the translational metrics we present is to provide an interpretation of these analytical metrics. The optimal translational metrics are by nature relatively arbitrary. As previously stated, to enhance understanding and the value of public health messages, it is useful to express recommendations in terms of time spent in common activities, e.g. brisk walking. To avoid the main problem with the cut-point approach, i.e. collapsing data into categories for analysis which renders it impossible to compare datasets deploying different cut-points, we turn cut-point analysis on its head. Instead of reporting the minutes above a given acceleration threshold, we report the minimum acceleration achieved for a given duration—MX where *X* refers to the duration, e.g. M60 refers to the minimum acceleration for the most active 60 min of the day. This acceleration can then be interpreted using cut-points, e.g. 200 m*g* for MVPA [17], or the acceleration associated with a typical activity, e.g. brisk walking. Importantly, this is applied at the translation stage rather than the analysis stage meaning that the data are not tied to a cut-point but can be interpreted using any cut-point. This is akin to the calculation of body mass index identically for adults and children, but interpretation with population-specific thresholds for overweight and obesity. Alternatively, pattern recognition approaches could be used to identify types of activities. However, these more sophisticated approaches are not currently widely accessible. The reason cut-point approaches are so widespread is their simplicity. We propose these metrics as an alternative, simple to apply, data-driven approach that avoids rendering datasets incomparable by applying cut-points prior to data analysis. It could enable meaningful visual comparisons of within- and between-group comparisons and generation of data-driven norms, and aid public-health friendly interpretation of data analyses carried out on 24-h data. Further, it uses the freely available open-source R package, GGIR [18], thus could be widely applied to enhance the translation of accelerometer data.

#### Translational metrics

The acceleration above which a person’s most active X minutes/time (MX) are accumulated focuses on a person’s most active periods rather than summarising the 24-h profile [19]. The active minutes can be accumulated in any way across the day, with no need for the activity to be in bouts in line with recent guidelines [20]. For example, if a child had an M60 value of 210 m*g*, this would indicate that the child accumulated 60 min of activity at accelerations (intensity) greater than 210 m*g* across the day. In the future, we may be able to compare this to accelerometer-driven physical activity guidelines (e.g. [21]). For now, for illustrative purposes, if we compare our M60 value to an MVPA cut-point, e.g. 200 m*g* [17], we can conclude the child is meeting the 60-min daily MVPA guideline [20, 22] as the M60 of 210 m*g* is higher than the cut-point. However, according to a more stringent 250-m*g* MVPA cut-point [23], the child does not quite reach the guideline as the M60 of 210 m*g* is lower than the more stringent cut-point. If we had followed the conventional cut-point approach, we would have collapsed our data upfront according to the 200-m*g* cut-point, i.e. each data-point simply classified as above or below 200 m*g*. This allows the calculation of the minutes accumulated above 200 m*g* but precludes comparison to any other cut-point. Importantly, with the MX approach, the data are not collapsed, but instead simply compared with a cut-point post-hoc. The advantages of post-hoc translation of accelerometer metrics are clear; we maintain the continuous nature of our variable, and we can compare the MX to any cut-point or acceleration indicative of a standard activity (e.g. brisk walking). In contrast, analysing data with cut-points imposes thresholds on the data, which are collapsed into categories for analysis, rendering it impossible to compare datasets deploying different cut-points [19].

Translating the MX metrics in terms of indicative activities (e.g. brisk walking and running) provides a public-health friendly interpretation. Using a broad range of MX variables provides a meaningful illustration of the intensity and volume of the 24-h activity profile, facilitating comparisons between and within groups. For example, the M120, M60, M30, M15 and M5 illustrate the more active periods of the day, while the M^1^/_3DAY_ refers to the most active ^1^/_3_ (8 h) of the day. The value of the M^1^/_3DAY_ is thus the acceleration that discriminates between the least and most active half of the waking day, if sleep approximates 8 h. These are exemplar MX metrics, more or fewer could be used, but we have found that six to eight (adding the M10 and M2 to the metrics above) MX metrics provide a good illustration of the activity profile. To ensure comparability between datasets, it would be necessary for agreement on the durations of the MX metrics to be presented. We speculate that it would perhaps be easier for researchers to agree on a range of time durations of interest that could be applied to all populations than it would be to agree on specific intensity cut-points which not only vary within and across populations but are also dependent on the protocol used to generate them. In the following sections, we provide examples of how the translational metrics can be used to interpret and translate results obtained from applying the analytical metrics to several diverse samples of children and adults. All samples wore GENEActiv or ActiGraph raw acceleration wrist-worn accelerometers 24 h a day for up to a week [14]; data were processed in the open-source R package, GGIR [18], as previously described [14]. In brief, the average magnitude of dynamic acceleration corrected for gravity (Euclidean Norm minus 1 *g*, ENMO) was averaged over 5 s epochs expressed in milli-gravitational units (m*g*). Participants were excluded if they had fewer than three days of valid wear (defined as > 16 h per day), or wear data was not present for each 15-min period of the 24-h cycle. The default non-wear setting was used, i.e. invalid data were imputed by the average at similar time-points on different days of the week. The average acceleration, intensity gradient and MX metrics were generated for each day and averaged across all valid days.

### Examples of translation

#### Between-group comparisons

**Fig. 1:**
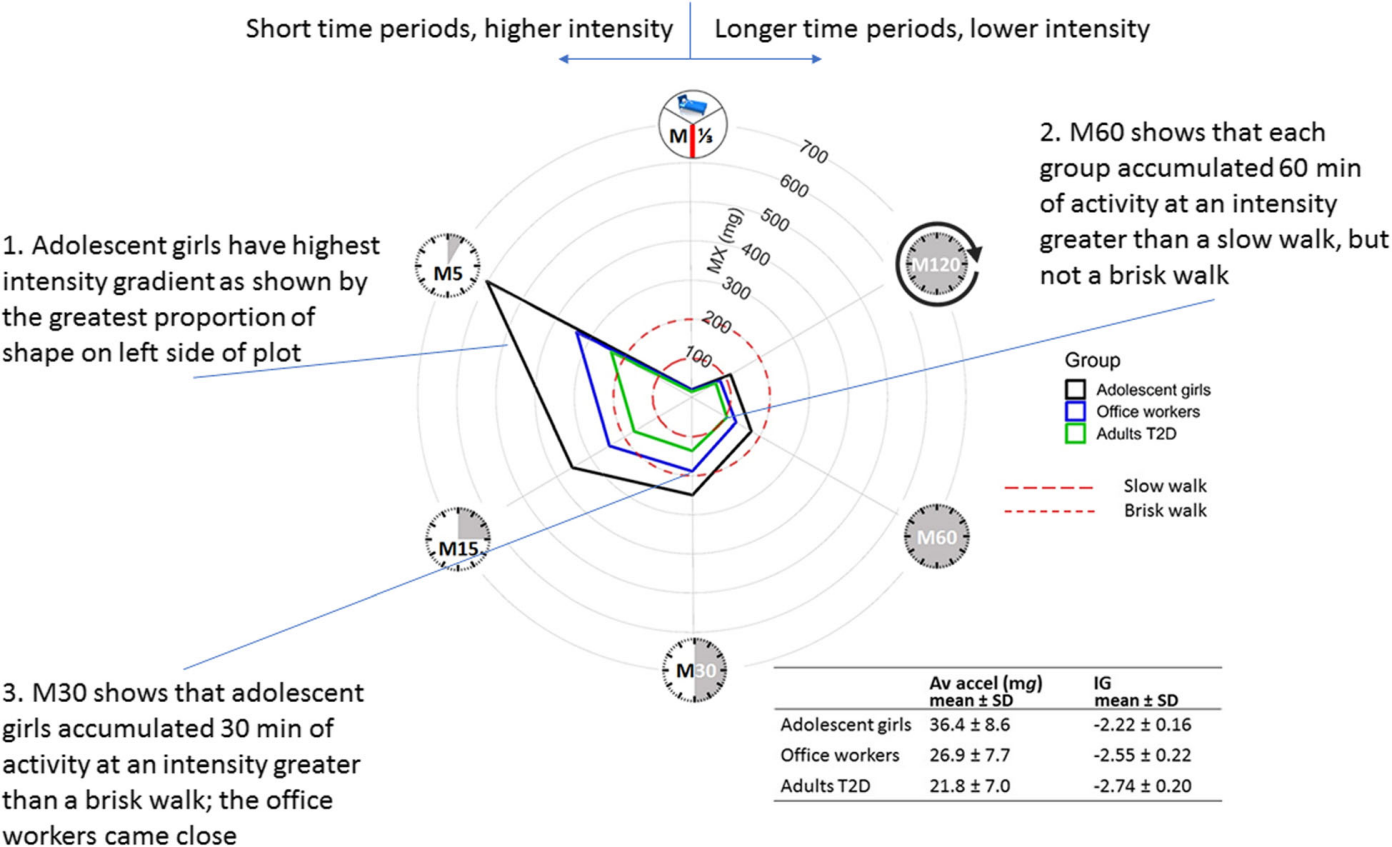
Radar plot illustrating MX metrics for (clockwise) the most active 8 h of the day (M^1^/_3DAY_), 120 min (M120), 60 min (M60), 30 min (M30), 15 min (M15) and 5 min (M5) for three groups with differing average acceleration and intensity gradient: highest: adolescent girls, mid: adult office workers, lowest: adults with type 2 diabetes. Av accel, average acceleration; IG, intensity gradient; T2D, type 2 diabetes; SD, standard deviation

Plotting the MX metrics on a radar plot illustrates the activity profile quantified by the analytical metrics, the intensity gradient and average acceleration; tables of exact values for means and standard deviations for the MX values could be provided in supplementary information of manuscripts. Figure 1 shows the MX metrics for three groups differing in both average acceleration and intensity gradient: highest: adolescent girls (*N* = 1669, age (mean ± SD) = 12.8 ± 0.8 years), mid: adult office workers (*N* = 114, age = 41.2 ± 10. 9 years), lowest: adults with type 2 diabetes (*N* = 475, age = 64.2 ± 8.7 years) [14]. The MX metrics are plotted on the radii of the plot, one radius for each metric. The points are joined, resulting in a shape for each group; the greater the surface area of the plotted shape on the left of the radar plot (where the shorter duration MX metrics (M5, M15, M30) are situated) is, the higher the intensity gradient. This is highest for the adolescent girls, followed by the office workers, then the adults with type 2 diabetes. The dashed red circles show approximate accelerations associated with a slow and brisk walk [14, 17, 19, 23, 24]; these can be used to translate the MX metrics. For example, the average M60 for each group is indicative of intensities greater than a slow walk for 60 min per day, but not a brisk walk. The average M30 for the adolescent girls is indicative of intensities greater than a brisk walk for 30 min per day, but not for the other groups, although the office workers come close. These radar plots provide an informative illustration of the intensity profile associated with differing intensity gradients allowing diverse populations to be compared directly. The dashed red lines enable the translation of the MX metrics in terms of time spent in typical activities, e.g. slow and brisk walking, providing meaningful interpretation. These are ballpark estimates based on calibration studies and energy expenditure compendiums as described in Rowlands et al. [19]. Currently, there are limited data from which to draw these estimates (e.g. [17, 23, 24, 25) and there is a need to generate more data showing the acceleration ranges associated with representative activities across a wide range of demographics.

#### Meeting guidelines: example with adolescent girls

**Fig. 2:**
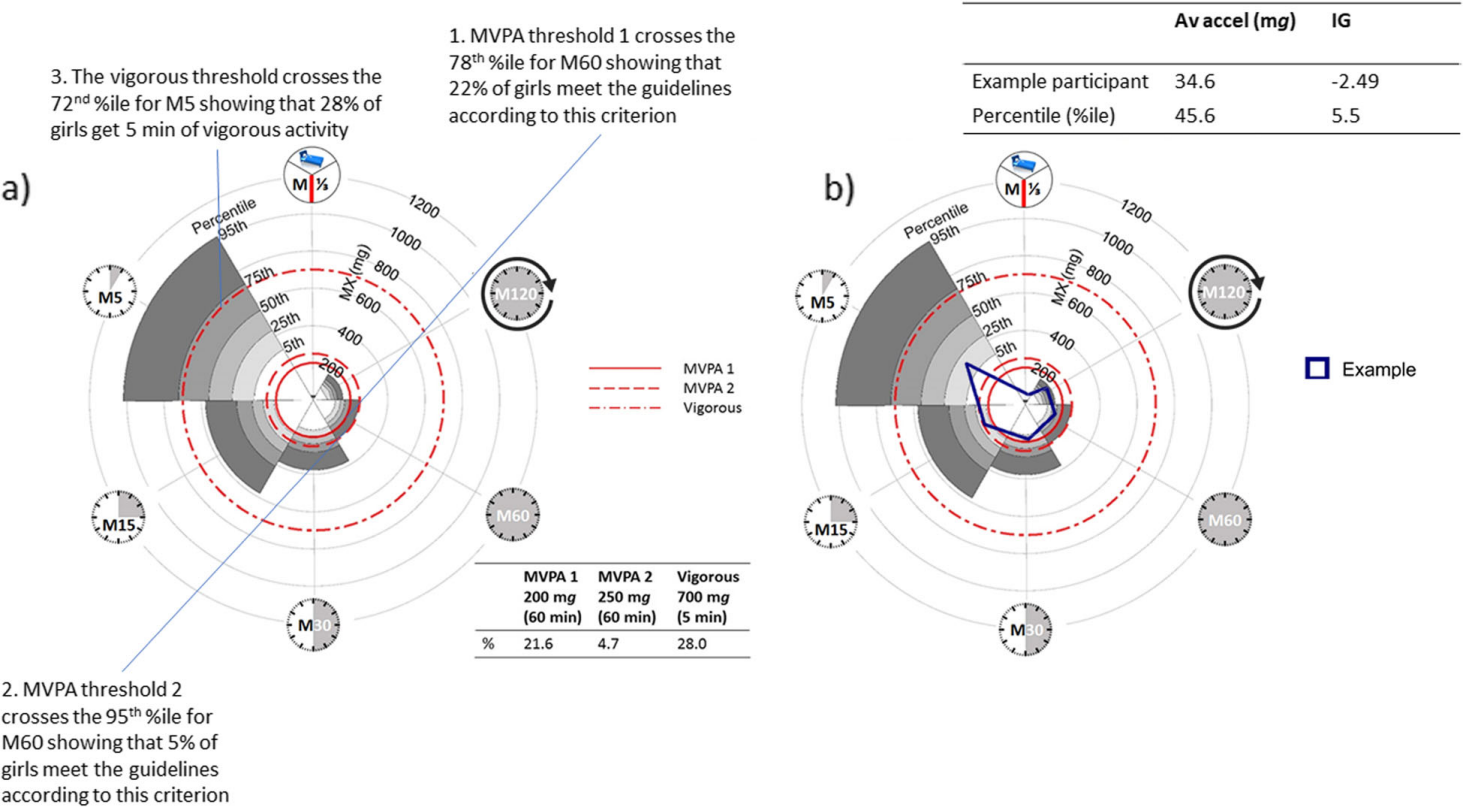
**a** Evaluation of meeting guidelines (adolescent girls): Presenting percentiles for (clockwise) the most active 8 h of the day (M^1^/_3DAY_), 120 min (M120), 60 min (M60), 30 min (M30), 15 min (M15) and 5 min (M5) facilitates evaluation of meeting guidelines relative to any given threshold. Examples are shown for two MVPA thresholds. **b** Comparison of a single participant (14-year-old overweight girl) relative to the sample percentiles. The longer duration MX metrics are relatively high or comparable to the sample (M^1^/_3DAY_ = 72nd percentile, M120 and M60 > 47th percentile), while the shorter duration MX metrics reflect low-intensity activity relative to the sample (M30, M15 and M5 < 38th percentile). Together this results in similar average acceleration to the group median, but much lower intensity gradient. Av accel, average acceleration; IG, intensity gradient; MVPA, moderate-to-vigorous physical activity

It is possible to look at a single group more closely. Moving forward, it is desirable to develop accelerometer-driven physical activity guidelines [21, 25], rather than inappropriately comparing physical activity assessed by accelerometer cut-points to guidelines developed from self-report data, which are conceptually different [26]. But presently, for example, the M60 metric can be used to assess whether children are meeting current MVPA guidelines. Figure 2a plots the percentiles for each MX metric for our sample of adolescent girls facilitating evaluation in terms of cut-points or normative acceleration values typical of walking/running. The grey shading for each MX metric represents the percentile with the lowest percentile in white through to the highest in dark grey. The levels for each percentile are marked on the M5 metric. While the M60 shows that 22% of girls meet the guidelines according to MVPA threshold 1 [17], this drops to 5% for MVPA threshold 2 [23]. It can also be seen that 28% of girls obtain 5 min of vigorous activity [17]. Comparison to any cut-point/typical activity is possible. It is difficult to see the results for the longer duration MX metrics; we are working towards developing interactive online plots that will allow readers to expand parts of the radar plots and to obtain exact values for the MX metric by hovering the mouse over the plots. The use of companion plots showing the standardised MX metrics can also be used to clearly illustrate differences in metrics of all durations as demonstrated in the ‘Within-group comparisons, illustrating differences in intensity gradient’ section.

The MX metrics for sub-groups or individual participants can be overlaid to illustrate the percentiles each MX metric falls on. For example, Fig. 2b overlays the MX metrics for an overweight 14-year-old girl. The average acceleration for this girl was near the median (46th percentile), while the intensity gradient was very low relative to her peers (6th percentile). The intensity of the longer duration MX metrics is relatively high or comparable (M^1^/_3DAY_ = 72nd percentile and M120 = 55th percentile, M60 = 47th percentile, right-hand side of the plot), while the shorter duration MX metrics reflect low-intensity activity relative to the whole group (M30 = 38th percentile, M15 = 28th percentile, M5 = 16th percentile, left side of plot). The comparatively high levels of M^1^/_3DAY_ and M120, indicating more ‘pottering around’, result in similar average acceleration to the median (46th percentile) despite relatively little high-intensity activity. This participant spends about 30 min in MVPA (threshold 1, 15 min according to threshold 2). Moving forward, population-referenced age- and sex-specific percentiles could be generated for the analytical and translational metrics allowing us to interpret physical activity levels as we do body mass index (BMI), fitness, height and weight.

#### Within-group comparisons: illustrating differences in intensity gradient

**Fig. 3:**
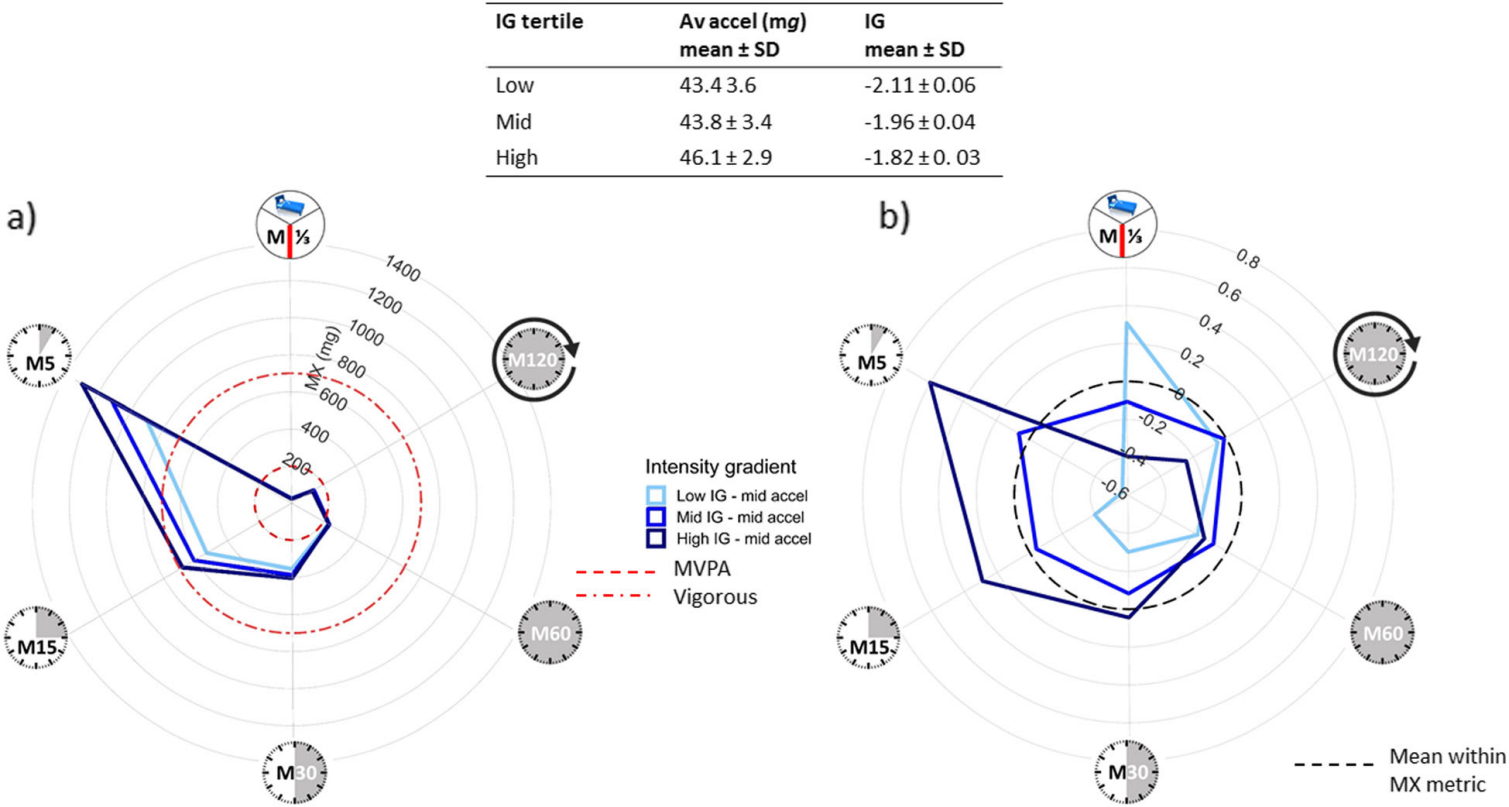
Translation of negative association between intensity gradient and BMI, independent of average acceleration (10-year-old children). Illustration of the physical activity profile associated with low/mid/high-intensity gradient, but similar average acceleration for **a** raw MX metrics ((clockwise) the most active 8 h of the day (M^1^/_3DAY_), 120 min (M120), 60 min (M60), 30 min (M30), 15 min (M15) and 5 min (M5)) and **b** standardised MX metrics. As the MX metrics are standardised within metric the mean = 0 (dashed black line) and SD = 1. The standardised plot (**b**) for children with the lowest intensity gradient is skewed to the right due to high levels of M^1^/_3DAY_ and M120; this results in similar average acceleration despite low levels of M5, M15 and M30. BMI, body mass index; SD, standard deviation; Av accel, average acceleration; IG, intensity gradient; MVPA, moderate-to-vigorous physical activity

While average acceleration was not associated with BMI in our sample of 10-year-old children (*N* = 145, age = 9.6 ± 0.3 years) [14, 27], the intensity gradient was independently negatively associated (*p* < 0.001). To translate the association with intensity of activity when the volume is similar, Fig. 3a illustrates the MX metrics for children with similar average acceleration (mid-tertile), but different intensity gradient (low/mid/high tertile). Plotting data for children with similar average acceleration, but low, mid and high-intensity gradient, focuses on the importance of intensity highlighted by the independent main effect of the intensity gradient. The higher intensity gradient is clearly seen in the proportionally greater areas covered on the left of the plot for the high- and mid-intensity gradient groups. This can be seen more clearly on a standardised plot, where the MX metrics are standardised within metric (Fig. 3b). As each MX metric is standardised, the mean is 0 (marked by the dashed black line) and the SD is ± 1. (Note, here the MX metrics are standardised within the sample, but when population-based norms are available it would also be possible to standardise them relative to norms.) Children in the lower intensity gradient tertiles show comparatively high levels of M^1^/_3DAY_ and M120 or ‘pottering around’, resulting in similar average acceleration despite relatively little high-intensity activity (low M5, M15, M30). It can also be seen that the intensity gradient tertiles are similar for the M60, but increasingly disparate for the M30, M15 and M5. On average, all groups get 60-min MVPA (M60 > 200 m*g*, Fig. 3a), but within this 60 min the highest intensity gradient group averages 15 min of vigorous activity (M15 > 700 m*g*, Fig. 3a) including 5 min of much higher intensity activity (M5 > 1200 m*g*, Fig. 3a). A similar pattern of results, albeit at lower intensity, was obtained for our sample of adolescent girls. These are cross-sectional data, but if further research supported these findings and a causal relationship was found, it would suggest that of the 60-min accumulated MVPA recommended daily for children at least 5-15 min should be higher intensity, e.g. 15 min jogging including 5 min of faster running/jumping.

#### Within-group comparison: illustrating differences across domains and/or time periods

**Fig. 4:**
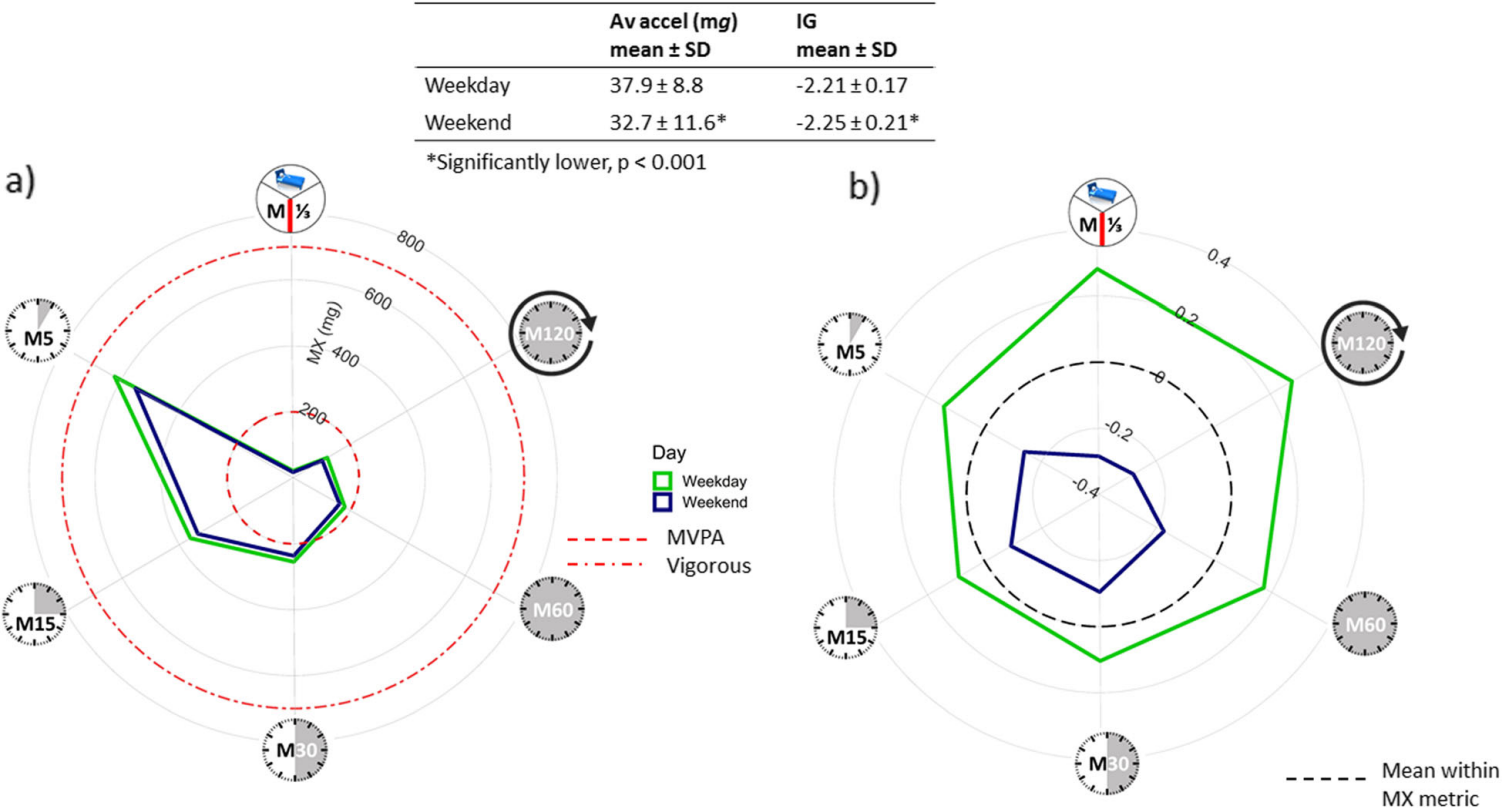
Activity profile illustrated with **a** raw MX metrics ((clockwise) the most active 8 h of the day (M^1^/_3DAY_), 120 min (M120), 60 min (M60), 30 min (M30), 15 min (M15) and 5 mins (M5)) and **b** standardised MX metrics across time periods: weekdays/weekend (adolescent girls). Average acceleration and intensity gradient were significantly lower on weekends than weekdays. As both volume and intensity of activity were lower all MX metrics, regardless of duration, were correspondingly lower. SD, standard deviation; Av accel, average acceleration; IG, intensity gradient; MVPA, moderate-to-vigorous physical activity

The plots can also be used to explore differences between domains, e.g. occupational/leisure, and time periods, e.g. weekdays/weekend. The activity profile of our adolescent girls showed significantly lower average acceleration and intensity gradient on weekends compared with weekdays. This is illustrated in Fig. 4a (raw plot) and 4b (standardised plot). As both volume and intensity of activity were lower on the weekend, it is not surprising that all MX metrics, regardless of duration, were lower. The relative differences were greatest for the longer duration low-intensity metrics (M120, M^1^/_3DAY_, Fig. 4b). This is in contrast to when the intensity gradient is lower but the average acceleration is similar (Fig. 3a, b), where the activity profile is characterised by relatively high M120 and M^1^/_3DAY_ coupled with relatively low M5, M15 and M30.

### *Application to different wear-sites*

**Fig. 5:**
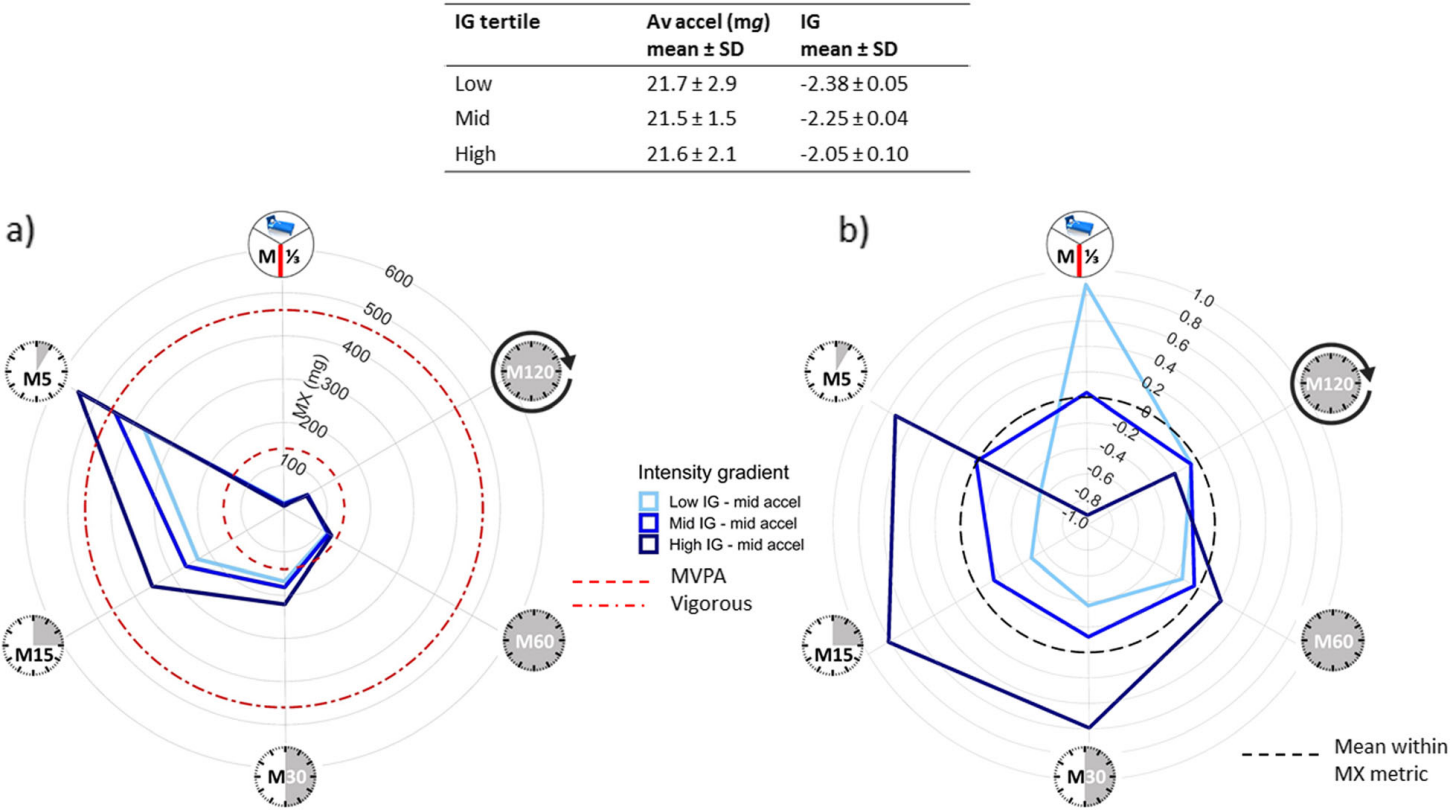
Application to different wear-sites: Data from hip-worn monitors (10–12-year-old children) translated in the same way as the children’s data from wrist-worn accelerometers in Fig. 3. **a** Raw MX metrics ((clockwise) the most active 8 h of the day (M^1^/_3DAY_), 120 min (M120), 60 min (M60), 30 min (M30), 15 min (M15) and 5 min (M5)) and **b** standardised activity profiles of children with similar average acceleration (mid-tertile), but low, mid and high-intensity gradient. The standardised plot (**b**) for children with the highest intensity gradient is skewed to the left due to high levels of M5, M15 and M30 resulting in similar average acceleration despite low levels of M^1^/_3DAY_ and M120. SD, standard deviation; Av accel, average acceleration; IG, intensity gradient; MVPA, moderate-to-vigorous physical activity

While the values of these analytical and translational metrics are wear-site specific, the same principle could be applied to monitors worn at any wear-site. To illustrate this, we applied the analytical metrics to data from accelerometers worn at the hip by 10–12-year-old children (*N* = 58) [28]. Consistent with results from children and adolescent girls wearing wrist-worn accelerometers [14, 16], the intensity gradient was independently negatively associated with BMI and percent fat (*p* < 0.05). Figure 5a (raw) and b (standardised) show that these data translated in the same way as the children’s data from wrist-worn accelerometers in Fig. 3. The dashed line shows hip site–specific cut-points for MVPA and vigorous activity [17]. On average, all intensity gradient tertiles attain 30 min of MVPA, but not 60 min, and only the mid and high tertiles attain 5 min of vigorous/running activity. Albeit cross-sectional, as with the data from wrist-worn accelerometers (Fig. 3), these data from hip-worn accelerometers suggest it may be prudent to consider including shorter periods (5–15 min) of running type activity within the 60-min MVPA daily guidelines.

**Fig. 6:**
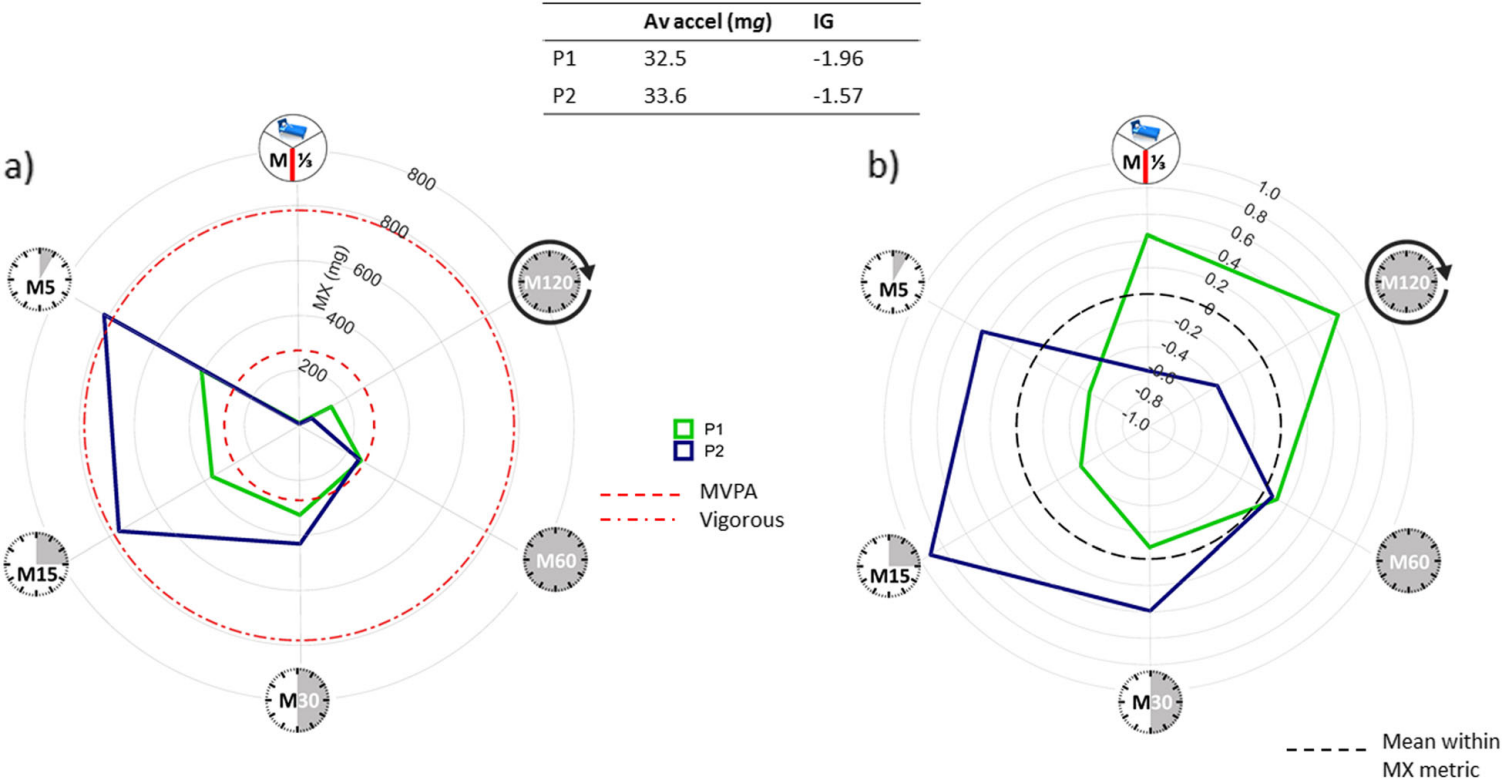
Application to different wear-sites. Data from thigh-worn monitors. **a** Raw MX metrics ((clockwise) the most active 8 h of the day (M^1^/_3DAY_), 120 min (M120), 60 min (M60), 30 min (M30), 15 min (M15) and 5 min (M5)) and **b** standardised activity profiles for two example adult participants (P1 and P2) with similar average acceleration but differing intensity gradients. P1 has a low-intensity gradient, with comparatively high levels of M^1^/_3DAY_ and M120 (**b**), P2 has a high-intensity gradient characterised by a greater area on the left-hand side of the radar plot. This results in a similar average acceleration. Av accel, average acceleration; IG, intensity gradient; MVPA, moderate-to-vigorous physical activity

Figure 6a (raw) and b (standardised) plot unpublished data from our research group from thigh-worn accelerometers for two healthy adults (P1 and P2) with similar average acceleration but differing intensity gradients (*N* = 20, age = 28.3 ± 6.2 years). As with wrist-worn (Fig. 2) and hip-worn (Fig. 6) accelerometers, a high-intensity gradient (P2) is characterised by vigorous activity, with a greater area covered on the left of the radar plot (Fig. 6a), while the participant with the low-intensity gradient (P1) has comparatively high levels of M^1^/_3DAY_ and M120 (Fig. 6b), resulting in similar average acceleration despite relatively little high-intensity activity.

It is important to note that although these analytical and translational metrics can be applied to any wear-site, with the pattern of results appearing to be similar, the values of the metrics are wear-site specific. Therefore, the metrics should only be compared when collected at the same wear-site. In particular, while the example activity profiles from the hip and the thigh are based on small samples (hip) or example participants (thigh), they suggest the M5 value tends to be higher relative to the other MX metrics for wrist-worn monitors. Further, measured acceleration may differ between some brands of monitor; while values appear to be similar between the GENEActiv and Axivity devices, average acceleration values are approximately 10% lower for the ActiGraph [29]; it may be possible to address this with an affine conversion of the acceleration values. The examples herein use accelerometer data collected at 100 Hz and the average of the ENMO metric [18] over 5 s epochs as is widely used [14–17, 21]. However, the principle could be applied to any sampling frequency, epoch. Note that while the analytical and translational metrics should be relatively independent of the sampling frequency [30], the epoch should be similar between datasets being compared as averaging over larger epochs would smooth out higher intensity activity.

### Pros and cons of the proposed data-driven metrics for analysis and translation

#### *Pros*

- Easy to generate analytical and translational metrics that could be widely applied using open-source software, GGIR [18], that is already routinely deployed in epidemiological studies.
- Data-driven approach reflects directly measured acceleration and maintains the continuous nature of accelerometer metrics, minimising prediction error.
- Translational metrics can be interpreted post-hoc in relation to any cut-points, e.g. recently developed age-equivalent cut-points [31] and/or accelerations indicative of typical activities. This shifts prediction error to translation and facilitates the development of public-health friendly recommendations.
- As data accumulate, population-based norms could be developed for the analytical and translational metrics enabling researchers and clinicians to interpret physical activity profiles of samples and individuals in relation to norms.

#### *Cons*

- To ensure comparability between datasets, it would be necessary for agreement on key MX metrics to be presented.
- Metrics are wear-site specific and may differ between some brands of monitors. For example, evidence suggests that while the magnitude of acceleration measured by the GENEActiv and Axivity devices is similar, values are approximately 10% lower for the ActiGraph [29].
- The emphasis on a simple widely applicable approach means further information contained in the accelerometer signal, e.g. relating to angles and the frequency domain, is ignored and more sophisticated pattern recognition approaches are not used to identify types of activities.
- The metrics do not take into account the temporal pattern of accumulation. Incorporation of metrics such as the aggregate [32] and the most active continuous X minutes could be beneficial.

## **Conclusion**

The MX metrics facilitate a visual and public-health friendly translation of analyses conducted using the analytical metrics: average acceleration and intensity gradient. We have demonstrated how radar plots of the MX metrics can be used to provide (i) a visual translation of between- and within-group comparisons; (ii) information on meeting physical activity guidelines; (iii) visualisation of individual physical activity profiles relative to the larger group or norms (if available); and (iv) comparison of activity profiles between domains and/or time periods. Further, the MX metrics associated with differing intensity gradients, but similar average acceleration, illustrate the importance of high-intensity activity where independent and/or interactive associations of the intensity gradient are seen with health markers [14]. The MX metrics associated with ‘healthy’ physical activity profiles can be interpreted using accelerations indicative of typical activities to provide effective public health messages. This is a translational methodology, rather than a prescribed method; it could be applied to a variety of scenarios, some of which are exemplified here. While the values of the analytical and translational metrics are wear-site specific, the principle could apply to any wear-site.

## Data Availability

Adolescent girls: Owing to the use of opt-out consent, and not including any specific data sharing information in the participant and parent/guardians information sheets, there are no data that can be shared publicly. Other anonymised datasets reported during the current study are available on reasonable request. Requests for access to data should be addressed to the corresponding author at alex.rowlands@le.ac.uk. All proposals requesting data access will need to specify how it is planned to use the data, and all proposals will need the approval of the appropriate trial co-investigator team before data release.

## Abbreviations

M^1^/_3DAY_: The acceleration above which a person’s most active ^1^/_3_ (8 h) of the day is accumulated.
MVPA: Moderate-to-vigorous physical activity
MX: The acceleration above which a person’s most active X minutes/time are accumulated
